# Prisoners with reduced criminal responsibility stand out based on their rates of hospitalisation during their sentences

**DOI:** 10.1108/IJPH-05-2022-0032

**Published:** 2023-07-25

**Authors:** Miisa Törölä, Mika Rautanen

**Affiliations:** UEF Law School, University of Eastern Finland, Joensuu, Finland; Health Care Services for Prisoners, Finnish Institute for Health and Welfare, Helsinki, Finland

**Keywords:** Prisoners, Health services, Prison, Hospitalisation, Reduced criminal responsibility, Universal health care system

## Abstract

**Purpose:**

Globally, health problems are very common among prisoners. A mental state examination aims to help in recognising psychiatric problems among offenders and the possible association of these psychiatric issues with their committed crime. The legal-medical term “reduced criminal responsibility” refers to a weakened sense of reality and the ability to control one’s behaviour because of compromised mental health and without an evaluated need for forensic psychiatric hospitalisation. However, little is known about the actual need for the health care of prisoners with reduced criminal responsibility (PRCR). The purpose of this study was to explore treatment-related visits to prison by PRCR in Finland.

**Design/methodology/approach:**

The research data comprise information on PRCR’s treatment-related visits and that of a matched control group (*n* = 222). Descriptive cross-tabulation with X²- and nonparametric Mann–Whitney U-tests and Cox regression analyses are applied.

**Findings:**

The results show that almost every PRCR had at least one treatment-related visit during their sentences. Visits to a psychiatric hospital for prisoners, to the prison hospital and especially to a civil hospital are more common among PRCR. The need for treatment appears significantly earlier in their sentences.

**Originality/value:**

These findings demonstrate the PRCR’s greater need for access to health services and the need for further development between the Health Care Services for Prisoners, Prison and Probation Service of Finland and public health and social services in Finland. More exploration of the medical reasons and locational distribution of the vast amount of civil hospitalisation is needed.

## Introduction

In Western societies, prisoners are more likely to experience health problems than the general population [Butler *et al.*, 2006; Condon *et al.*, 2007; Fries *et al.*, 2013; *Joukamaa et al*., 2010; Morthorst *et al.*, 2021; Sirdifield *et al.*, 2009; Watson *et al.*, 2004; World Health Organization (WHO), 2005]. The discrepancies in health and in the usage of health care services among Finnish inmates have been studied based on gender and type of incarceration (Joukamaa *et al.*, 2010) but not based on reduced criminal responsibility, which usually indicates mental health problems.

The current study explores the quantity and timing of the realised hospitalisations of prisoners with reduced criminal responsibility (PRCR) in Finland. Reduced criminal responsibility is one of the three decision categories – *criminal responsibility*, *reduced criminal responsibility* and *not criminal responsibility* – in Finnish criminal law. In most cases, a decision on reduced criminal responsibility is made based on a forensic psychiatric examination. In these cases, the perpetrator of a crime is remanded for forensic psychiatric examination by the court. During the evaluation process, the offender’s ability “to understand the factual nature or unlawfulness” of their criminal act or control their behaviour is evaluated as not being crucial – even though it is “significantly weakened” – at the time of the criminal act because of “mental illness, severe mental deficiency, mental disturbance or a disturbance of consciousness” (Criminal Code of Finland, chapter 3, section 4). Offenders with reduced criminal responsibility are usually convicted to imprisonment [1]. Although the number of offenders convicted because of reduced criminal responsibility has decreased in recent decades, around 10 people are convicted annually (Lappi-Seppälä and Kolehmainen, 2021, p. 32).

Mental state examinations serve the purpose of distinguishing the most severely psychiatrically ill offenders and excluding them from the prison population. An offender with reduced criminal responsibility – and hence serving a sentence in prison – is nevertheless not placed in a special prison unit or directed to health care services based on the mental state examination. It is acknowledged that a mental state examination almost always results in some type of diagnosis being made. Offenders evaluated as criminally responsible and with reduced criminal responsibility have a similar diagnostic profile. Over the past 30 years, the most visible difference in diagnoses, on average, is a slight increase in the number of psychoses in the PRCR group (Lappi-Seppälä and Kolehmainen, 2021, p. 49). To develop the criminal sanction system into more appropriate for their needs, it is important to explore to what extent PRCR use health services and whether their need for health services is different from other inmates.

The current article is organised as follows: First, we describe the Finnish health care service for prisoners. After that, we will continue to elaborate on prisoners’ health issues and their usage of health services, as well as the factors contributing to health care help-seeking behaviours. The empirical part of the article will focus on a comparison of treatment-related exits from prison between the PRCR and control groups (CGs). The article then proceeds to a discussion of the findings and their indications for practice and research on prison health care services.

## Health care services for prisoners in Finland

The universal health care system and normality principle that are abided by the Finnish criminal sanction system mean that prisoners have the same rights to services as other members of society, with the distinction that they cannot choose health care service providers themselves. The health care system for Finnish prisoners, including primary and oral health care and specialised psychiatric medical care, is provided by the Health Care Services for Prisoners (VTH) (Laki vankiterveydenhuollon yksiköstä 1635/2015; Imprisonment Act 767/2005, chapter 10; Remand Imprisonment Act 768/2005, chapter 6). The VTH operates under the Finnish Institute for Health and Welfare (THL) and in cooperation with the Prison and Probation Service of Finland (RISE). In practice, outpatient and oral health care clinics are situated inside prisons. Acute psychiatric treatment for prisoners is provided by the psychiatric hospital for prisoners, with two units located in the west and south of Finland. Additionally, a prison hospital located in southern Finland provides treatment and rehabilitation for somatic illnesses. In cases when the VTH’s services are not available, inmates are taken to the nearest civil health centre (Heikkinen *et al.*, 2022).

The duration of access to health care is legislated in the Health Care Act (1326/2010, sections 51–52). When the prisoner contacts the VTH’s primary care provider, the need for treatment must be assessed within three days. Furthermore, the treatment must be provided within three months of the assessment. Additionally, after receiving notification, an assessment of the need for psychiatric specialised care should begin within three weeks in the specialised care unit (VTH, 2022). Visits to health care are regularly documented and registered in health units, both in prison and civil units. However, patient information does not automatically transfer between civil health services and VTH because of incompatible patient information systems; rather, the flow of information depends on patients’ consent and activities to forward it. Additionally, the findings of mental state examinations are intended only as a basis for the decision of the court and, hence, are not documented in the patient information system without patients’ consent (Ahlgrén-Rimpiläinen *et al.*, 2021; Heikkinen *et al.*, 2022).

In prison, psychiatric services include 54 hospital beds serving about 3,000 daily inmates, and the somatic hospital has 34 beds. In general, for the Finnish population of 5.5 million, there are 2,600 psychiatric beds. This general number has declined from 22,000 in the 1970s (Linnaranta, 2022). In contrast, the number of prisoner beds has remained at the same level. Regardless of the proportionally high level of resources, prison services cannot meet the demands of continuous and systematic patient care because the sentences tend to be short and there often is insufficient coordination with after-release public health and social services. In a thorough assessment of prison health care services, this lack of bridging treatment plans was seen as a key weakness (Junnila, 2018, p. 41).

Psychological services operate as part of the social services in prison and are available to all inmates. In practice, psychologists work mainly in closed prisons (higher security-level units).

### Mental health and substance rehabilitation in prisons

In the Finnish criminal sanction system, substance abuse is acknowledged as a factor of reoffending, whereas mental health problems (leading t*o criminal responsibility* or *reduced criminal responsibility*) are not (Lappi-Seppälä, 2000, pp. 71–72). This may be a contributing factor to the different practical constructions of the interventions concerning substance abusive behaviour and mental health symptoms.

The need for substance abuse intervention or use for mental health services is assessed by the criminal sanction officers, who are responsible for compiling the sentence plans. With the exception of the opioid substitution treatment and general detoxification treatments provided by the VTH, all rehabilitating substance abuse interventions are arranged mainly as part of the social services provided by the prison units, whereas mental health interventions are part of the health services (Tourunen *et al.*, 2012, p. 580). This division has a twofold effect on mental health rehabilitation in prisons. First, social services in prison may not have compiled information on prisoners’ health-related rehabilitation needs and, hence, may not provide suitable interventions for prisoners with mental health issues [the lack of mental health-related interventions; see Rikosseuraamuslaitos (RISE) 2021]. Second, the prisoners’ active role in seeking help with health issues during their sentences is emphasised.

Another issue relating to accessibility, particularly to mental health services, is the geographical location of prison units. For example, the distance between Pelso prison (located in the northern part of Finland) and the psychiatric hospital for prisoners – the Turku unit – is approximately 600 km. In general, the longer the distance to the nearest health centre, the more time and resources are required for prisoner transfer (Suistomaa, 2014, p. 42).

## Prisoners’ need for and usage of health services

The most common health issues of inmates are related to mental health, substance abuse and communicable diseases (Joukamaa *et al.*, 2010; Watson *et al.*, 2004). In addition, the majority of Finnish prisoners have poor oral health (Vainionpää *et al.*, 2017). According to the latest research on the health of Finnish criminal sanction clients (prisoners and community sanction clients), over half of the clients take at least one regular medication. Four out of 10 take medication for insomnia or mental disorders. About a third have been hospitalised in a psychiatric unit, and well over half had contact with an outpatient clinic prior to their sentence (Joukamaa *et al.*, 2010). Female prisoners and prisoners whose incarceration is because of defaulting on a fine particularly stand out as a group with poor somatic and mental health, traumatic experiences and the inability to work (Joukamaa *et al.*, 2010; Salisbury and Van Voorhis, 2009; Stanton and Rose, 2020; Viitanen *et al.*, 2012; Viitanen *et al.*, 2011). According to a recent study, the hospitalisations of the PRCR in the time of their sentences are more frequent and longer lasting compared with other prisoners, which indicates a more general identification and prevalence of all kinds of health ailments (Törölä, 2021).

There has been an increase in both multiple substance abuse problems and various mental disorders in the past three decades among Finnish inmates (Jüriloo *et al.*, 2017; Lintonen and Joukamaa, 2013; Lintonen *et al.*, 2012; Lintonen *et al.*, 2011). As many as 9 out of 10 prisoners have substance abusive behaviour. A similar proportion have mental health problems, which are often behavioural or mood related (Joukamaa *et al.*, 2010, p. 46). These figures are extremely high compared with other prisoners in the USA (Lintonen *et al.*, 2011, p. 447) and in other Nordic countries (Bjørngaard *et al.*, 2009; Giertsen *et al.*, 2015, p. 149; Nesset *et al.*, 2011). According to Elenij (2021), substance abuse among PRCR is at a surprisingly low level: only 16% have a substance abuse diagnosis. The most common diagnoses are personality disorders (36%), musculoskeletal disorders (22%), anxiety (18%), mood disorders (16%) and psychotic disorders (12%). However, these findings may reflect the way prison doctors are used to documenting diagnoses in VTH medical patients’ records.

## Health care help-seeking behaviour in prison settings

As a form of looking for support and health services, health care help-seeking behaviour depends on various psychological and practical terms.

*A prisoner will not seek help if they do not recognise any need for it.* In a Finnish prison health survey, about half of the respondents felt they were doing well when asked about their own understanding of their mental health and balance. A clinical assessment identified a mental health problem in about three out of four inmates (Joukamaa *et al.*, 2010, p. 35, 46). This suggests that there is a gap between the subjective and clinical assessments of prisoners’ mental health/well-being. At the same time, the higher the perceived level of psychological anxiety, the more help is sought, for example, for emotional imbalance or feelings of fear (McGrath *et al.*, 2020).

*Demographic features seem to matter differently in different criminal sanction systems.* In particular, female prisoners stand out as a group that consumes extensive health services in Finland (Viitanen *et al.*, 2013), whereas in Norway, there is no difference by gender (Nesset *et al.*, 2011). According to a study conducted in New Zealand, on average, older and more educated inmates sought psychological services more often than other prisoners (Skogstad *et al.*, 2006). Again, a Norwegian study (Nesset *et al.*, 2011) suggests that older prisoners tend to seek health care help more frequently than the younger prisoners, but educational level does not have the same kind of significance.

*The prison environment affects the motivation to seek help in various ways.* A UK study exploring young prisoners’ barriers to accessing psychological services finds that inmates’ perceptions of what others might think and the possibility to seem vulnerable or as “a snitch” can inhibit their help-seeking. In addition, an essential element of motivation for service use is trust. Distrust towards mental health work specialists and the fear that personal information could spread among prison staff are strong barriers in attempting to contact mental health services. Disbelief that mental health workers really care, feelings of isolation and powerlessness and experiences of services of the wrong kind may lower motivation as well (McGrath *et al.*, 2020; see also Howerton *et al.*, 2007; Mitchell and Latchford, 2010).

*Previous positive experiences of service use lower the threshold for seeking help.* Experiences of being heard increase the desire to contact health services when a need arises (Howerton *et al.*, 2007; Skogstad *et al.*, 2006; see also Morgan *et al.*, 2007; Nesset *et al.*, 2011).

## The present study

In the present study, we examine the quantity and timing of the realised hospitalisations of the PRCR in Finland.

The research questions are as follows:

RQ1. To what extent (how often and for how long) are PRCR committed to treatment compared with other prisoners?

RQ2. At what point in imprisonment does the need for treatment appear in groups of PRCR and other prisoners?

The hypothesis is that PRCR differ from their psychiatric status, such that the need for psychiatric hospitalisation is greater for this population.

## Data and methods

### Data

This case control study applies register-based data from the National Prisoner Database in Finland (VATI). The VATI covers information about Finnish inmates and the measures taken towards them during their sentences. The database does not contain inmates’ health information or information on mental state examinations. The aforementioned data are administrated by the VTH and THL databases, respectively. Because of the uncertainty about the records of out-of-prison treatment visits, the VATI is a more accurate source than the VTH database when looking at treatment-related exits from prison. The personal data of individuals who have been assessed as having diminished criminal responsibility between 2004 and 2017 from the THL are combined with information on incarceration terms after the mental health examination from the VATI.

The total number of persons charged with an offence and whose mental state were ordered to be examined by the court and evaluated with reduced criminal responsibility between 2004 and 2017 totals 200. When the identification data from THL are combined with VATI, 135 records of the offenders coinciding with the date of the mental state evaluation have been found in the VATI. Cases whose imprisonment is still continuing at the time of data collection (August 2020) have been excluded. The CG of inmates is collected from a pool of offenders imprisoned between the years 2000 and 2019 (*N* = 48,527).

The data comprise information on the incarceration of the PRCR and CG of inmates (*N* = 222). The ratio of the number of cases and control subjects is 1:1. In the data collection, a genetic matching algorithm for R (Sekhon, 2011) is applied to obtain a CG without an evaluation of reduced criminal responsibility. The control subjects drawn from the VATI control pool do not represent a typical Finnish inmate group but instead have a similar inmate profile as offenders with reduced criminal responsibility. The control data have been collected randomly and to be similar in terms of violent principal offence [2], gender, release from closed prison, foreign citizenship, the length of imprisonment, the current number of the conviction, the year of the imprisonment and the prisoner’s age at the end of the imprisonment (presented in Table 1).

The data about the time and destination of the inmates’ outside visits contains 25,119 entries in total, including returns to prison. The number of recorded statuses varies from 2 to 1001 per prisoner, meaning that some prisoners have had only one while others have had multiple visits outside prison. The number of treatment-related exits from prison is 1,276 and has been entered into 176 prisoners’ records.

### Variables

Treatment-related exits are indicated in the VATI by one of the following terms:

- “Treatment in another prison” principally refers to a prison hospital in southern Finland. It may also refer to health care in another prison unit or psychiatric hospital treatment for prisoners at the Vantaa unit, which is provided by the VTH;
- “Psychiatric Hospital for Prisoners, the Turku unit” refers to psychiatric hospital treatment for prisoners at the Turku unit or Vantaa unit provided by the VTH;
- “Placed in an outside facility” refers to a placement in an out-of-prison facility for substance abuse rehabilitation; and
- “In a civil hospital” refers to a visit to a civil hospital or a dental care facility. It may also refer to a permanent placement in a forensic psychiatric hospital. Some visits classified as civil hospital visits may have been declared as “miscellaneous exit”, meaning that all civil hospital or dental care facility visits may not be included.

*The length of treatment-related exits* and *the timing of the need for treatment* are explored using the actual dates of the beginning of imprisonments and treatment-related exits.

### Analyses

The matching of the case and CGs, as described above, enables a comparison of the amount and length of the treatment-related exits by cross-tabulation with X^2^-tests and nonparametric Mann–Whitney U-tests. The timing of the first treatment-related exits is compared between the PRCR and CGs of other prisoners by applying Cox regression analysis. The statistical analyses have been conducted using IBM SPSS Statistics, version 27. The unit of observation for the analyses is a prisoner.

### Research ethics

The authorisation for the identification of PRCR has been applied for and received from the Finnish Institute of Health and Welfare. Authorisation for the use of VATI data for research purposes has been applied for and received from the Prison and Probation Service of Finland. The research complies with the Finnish National Board on Research Integrity’s (TENK) guidelines.

## Number of treatment-related exits

Among the PRCR group and the CG, 93% and 64% have at least one treatment-related exit from their prison unit during their sentence, respectively. The amount and percentages of PRCR and other prisoners hospitalised during their sentences are presented in Table 2. The rates of hospital visits between PRCR and other inmates differ significantly [3]. The PRCR group has been hospitalised more frequently in all treatment categories, save for placement in an outside facility. In particular, the services of the psychiatric hospital for prisoners are almost entirely used by the PRCR.

## Length of treatment-related exits

The lengths of the treatment-related exits range from less than 1 day to 991 days (SD 58.14). About half of the recorded exits are a day long (50.6%). The average number of days in:

- treatment in another prison;
- psychiatric hospital for prisoners in Turku; and
- an outside facility is greater in the CG than in PRCR group (see Table 3).

In particular, the length of psychiatric treatment in Turku in the CG stands out, suggesting that the hospitalisations of PRCR are more frequent and shorter than those of the CG.

In total, the differences in the length of treatment-related exits are statistically significant between PRCR and other inmates (U =121,291, *p* < 0.000). However, visits to (1) treatment in another prison (U = 11,000, *p* = 0.174), (2) psychiatric hospital for prisoners in Turku (U = 2,565, *p* = 0.78) and (3) an outside facility (U = 24, *p* = 0.668) have a similar length, whereas stays at civil hospitals have been distinctly longer among PRCR (U = 45,751, *p* < 0.000). The total number of days for treatment is 183 in the PRCR group and 48 in the CG (U = 2,067, *p* < 0.000; not shown in Table 3).

## Timing of the first treatment-related entries

The period of time between the beginning of imprisonment and first treatment-related exit ranges from less than 1 day to 1,883 days. Based on a Cox regression analysis of the treatment entries of each of the four categories separately and of any kind of treatment, the average timing of the first contact and hazard ratios is shown in Table 4.

**Figure 1:**
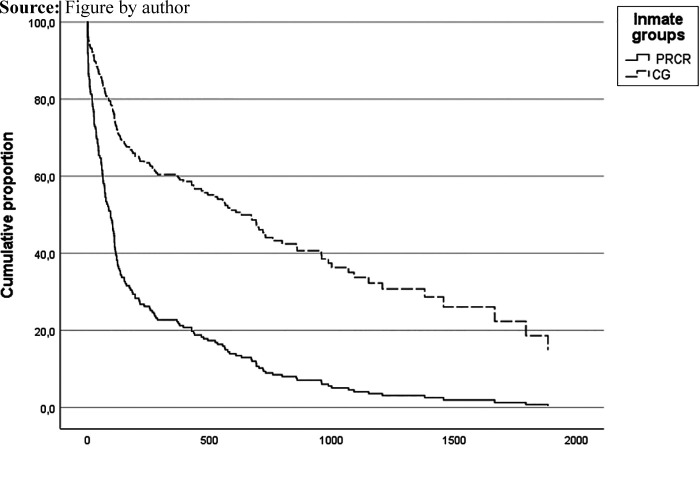
Cumulative proportion of the timing of the first contact with any kind of treatment of the PRCR and the control group in days

The Cox regression analysis reveals that PRCR are associated with a greater risk of hospitalisation to another prison, to psychiatric hospital for prisoners and to a civil hospital at an earlier point during their imprisonment periods. On average, the first treatment-related exit is to a civil hospital. Eleven of the PRCR entered any kind of treatment – treatment in another prison, psychiatric hospital for prisoners, an out-of-prison facility or in civil hospital – within the first 48 h of imprisonment. Half of the PRCR had a treatment-related exit within the first three months and 80% within the first year of their sentences (see Figure 1). In the CG, 20% had a treatment-related exit within the first three months and 36% within the first year of their sentences.

## Discussion

The present study has revealed that PRCR stand out by their rates of hospitalisation during their sentences. The hypothesis that PRCR have a greater need for psychiatric hospitalisation is confirmed. Visits to psychiatric hospitals for prisoners are substantially more common among PRCR. Psychiatric treatment periods are more frequent but shorter. Additionally, the number of entrances to the prison hospital/another prison care facility and to a civil hospital are more frequent in the PRCR group. Individual PRCR treatment periods are usually shorter, but overall, they spend more time in care facilities than in the CG. Furthermore, the need for treatment appears significantly earlier during their sentences.

The current study adequately represents the subpopulation of PRCR. The case control study design and matching method applied in data collection have enabled a comparison with prisoners without psychiatric evaluation of reduced criminal responsibility. The composition of the criminal-political system, including prisoners’ health services and rehabilitation, differs between countries. The division of criminal liability into *criminal responsibility*, *reduced criminal responsibility* and *not criminal responsibility* is not applied in all Western societies and not even in all Nordic countries. This narrows the generalisation of the results. Because of the slightly different coding habits between prison units, the VATI’s information about the purpose of treatment-related exits from the prison is not completely accurate. Some visits to civil hospitals may have been excluded from the data. In addition, the entries on visits to the Vantaa unit of the psychiatric hospital for prisoners are divided into two different categories of treatment visits. The data, therefore, impose restrictions on a more accurate exploration of treatment visits.

As a legal term, reduced criminal responsibility is controversial. Because of changes in legislation in the 2000s, the category does not have a straightforward effect on getting diminished prison sentences, nor does it require a treatment plan to be made. However, the present study shows that a mental state examination identifies the specific need for treatment when the offender is assessed as having reduced criminal responsibility. It may be that the identification of the need for health services by the VTH during the arrival inspection is adequate. Nevertheless, it is unknown whether the mental state examination as an intervention opens up new perceptions of one’s own state of health and the usefulness of adequate treatment for the examinees themselves.

The capacity and resources of the VTH to provide health care for prisoners are quite sufficient. However, relatively short sentences pose challenges for the provision of services. The VTH does not always have the time to form long-term medical treatment plans, and actualising these plans in the community after a person is released often seems difficult. Therefore, it is important that the need for care be considered in the sentence plans that use the expertise of the VTH, especially when deciding on placement in a prison unit. Prisoners who are in need of specialised care could be placed in a prison unit where the services are available on a daily basis. In addition, cooperation between RISE and the VTH and between the VTH and public health and social services varies between prison units. Efficient and well-structured practices for cooperation should be developed at the organisational level.

The rate of entering civil hospitals stands out as a very standard procedure for PRCR. The realisation of visits depends on the health status of the prisoners, their health care help-seeking behaviour and the availability of VTH services. Hence, future research can further explore both the medical causes for hospitalisations and actual geographical location of the PRCR to uncover the most probable reason for the differences in the usage of civil hospital services. Furthermore, the method used in the present study offers future possibilities for comparing other subgroups of prisoners and their needs for treatment and rehabilitation with the prison population.

## Acknowledgments

The authors would like to thank Senior Researchers Sasu Tyni and Marja-Liisa Muiluvuori (Finnish Criminal Sanction Agency) for their assistance with data collection and further consultation about the practicalities of the treatment-related entries. They would also like to thank Project Manager Pia Puolakka (Finnish Criminal Sanction Agency) for consultation about the psychological services available to inmates.

## Figures and Tables

**Table 1:** Distributions of the variables in the PRCR and CG (*N* = 222)

Background variable	PCRC	CG	All
Principal offence: violent (vs. nonviolent, %)	88.3	89.2	88.7
Gender: Male (vs. Female, %)	83.8	84.7	84.2
Release from closed prison (vs. open prison, %)	66.7	65.8	66.2
Foreign citizenship (vs. Finnish citizen, %)	4.5	3.6	4.1
The length of imprisonment (average; min–max)	1,053; 174–2,982	1,049; 177–2,983	1,051; 174–2,983
The current number of the conviction (average; min–max)	2.5; 1–21	2.6; 1–20	2.5; 1–21
The year of the imprisonment: (average; min–max)	2008; 2004–2017	2008; 2000–2017	2009; 2000–2017
Prisoner’s age at the end of the imprisonment (average; min–max)	41; 18–88	41; 17–81	41; 17–88

**Source**: Table by authors

**Table 2:** The number and proportion of PRCR and other prisoners hospitalised during their sentences (*N* = 174)

Prisoner group	Treatment in another prison**	Psychiatric hospital for prisoners, Turku***	Placed in an outside facility	A civil hospital***	Total***
PRCR, *n* (%)	54 (48.6)	59 (53.2)	5 (4.5)	94 (84.7)	103 (92.8)
CG, *n* (%)	32 (28.8)	9 (8.1)	4 (3.6)	61 (55.0)	71 (64.0)
Total, *n* (%)	86 (38.7)	68 (30.6)	9 (4.1)	155 (69.8)	174 (78.4)

Notes: **: *p* < 0.01;

*** *p* < 0.000

**Source**: Table by authors

**Table 3:** Average number of days in treatment for PRCR and the other prisoners

Prisoner group	Treatment in another prison	Psychiatric hospital for prisoners, Turku	Placed in an outside facility	A civil hospital***	Total***
PRCR	21	32	17	25	26
CG	28	80	22	3	13
Total	24	37	19	15	21

Note: ***p < 0.000

**Source**: Table by authors

**Table 4:** Hazard ratios from a Cox regression analysis on PRCR treatment entries within their imprisonment periods

		95.0% CI for Exp(B)		
Destination facility	Exp(B)	Lower	Upper	p-value
Treatment in another prison	2.199	1.414	3.419	<0.001
Psychiatric hospital for prisoners, Turku	8.942	4.429	18.055	<0.001
Placed in an outside facility	1.186	0.317	4.429	>0.05
A civil hospital	2.491	1.793	3.462	<0.001
Any kind of treatment facility mentioned above	2.945	2.147	4.040	<0.001

**Source**: Table by authors

## Notes

1. For more on the Finnish legislation and forensic psychiatric system, see Seppänen *et al.* (2020).

2. Offenders who are remanded for mental state examination have usually committed violent crime/s.

3. Treatment in another prison: *x*^2^(1) = 9.187, *p* < 0.01; psychiatric hospital for prisoners, Turku: *x*^2^(1) = 52.998, *p* < 0.000; a civil hospital: *x*^2^(1) = 23.280, *p* < 0.000; all hospitalisations: *x*^2^(1) = 27.218, *p* < 0.000. The statistical difference for the category “placed in an outside facility” is not estimated because of its small frequencies.
